# Deep sequencing reveals cell-type-specific patterns of single-cell transcriptome variation

**DOI:** 10.1186/s13059-015-0683-4

**Published:** 2015-06-09

**Authors:** Hannah Dueck, Mugdha Khaladkar, Tae Kyung Kim, Jennifer M. Spaethling, Chantal Francis, Sangita Suresh, Stephen A. Fisher, Patrick Seale, Sheryl G. Beck, Tamas Bartfai, Bernhard Kuhn, James Eberwine, Junhyong Kim

**Affiliations:** Department of Genomics and Computational Biology, Perelman School of Medicine, University of Pennsylvania, Philadelphia, PA USA; Department of Biology, School of Arts and Sciences, University of Pennsylvania, 301A/B Lynch Laboratory, 433 S University Avenue, Philadelphia, PA 19104 USA; Department of Pharmacology, Perelman School of Medicine, University of Pennsylvania, Philadelphia, PA USA; Current address: Allen Institute for Brain Science, Seattle, WA USA; Department of Pediatrics, Harvard Medical School, Boston, MA USA; Department of Cardiology, Boston Children’s Hospital, Boston, MA USA; Department of Cell and Developmental Biology, Perelman School of Medicine, University of Pennsylvania, Philadelphia, PA USA; Department of Anesthesiology, The Children’s Hospital of Philadelphia Research Institute, Philadelphia, PA USA; Harvard Stem Cell Institute, Cambridge, MA USA; Department of Stem Cell and Regenerative Biology, Harvard University, Cambridge, MA USA; The Department of Chemical Physiology, The Scripps Research Institute, La Jolla, CA USA; Current address: Richard King Mellon Institute for Pediatric Research, Children’s Hospital of Pittsburgh of UPMC, Pittsburgh, PA USA

## Abstract

**Background:**

Differentiation of metazoan cells requires execution of different gene expression programs but recent single-cell transcriptome profiling has revealed considerable variation within cells of seeming identical phenotype. This brings into question the relationship between transcriptome states and cell phenotypes. Additionally, single-cell transcriptomics presents unique analysis challenges that need to be addressed to answer this question.

**Results:**

We present high quality deep read-depth single-cell RNA sequencing for 91 cells from five mouse tissues and 18 cells from two rat tissues, along with 30 control samples of bulk RNA diluted to single-cell levels. We find that transcriptomes differ globally across tissues with regard to the number of genes expressed, the average expression patterns, and within-cell-type variation patterns. We develop methods to filter genes for reliable quantification and to calibrate biological variation. All cell types include genes with high variability in expression, in a tissue-specific manner. We also find evidence that single-cell variability of neuronal genes in mice is correlated with that in rats consistent with the hypothesis that levels of variation may be conserved.

**Conclusions:**

Single-cell RNA-sequencing data provide a unique view of transcriptome function; however, careful analysis is required in order to use single-cell RNA-sequencing measurements for this purpose. Technical variation must be considered in single-cell RNA-sequencing studies of expression variation. For a subset of genes, biological variability within each cell type appears to be regulated in order to perform dynamic functions, rather than solely molecular noise.

**Electronic supplementary material:**

The online version of this article (doi:10.1186/s13059-015-0683-4) contains supplementary material, which is available to authorized users.

## Background

The transcriptome is a key determinant of the phenotype of a cell [1] but increasing evidence suggests the possibility that large variation in transcriptome states exists across cells of the same type. High variability in single-cell transcripts have been described using various techniques, including targeted amplification [2–4], florescent in situ hybridization or FISH [5] and whole transcriptome assays [6–11]. In addition to variability in expression levels, RNA sequencing from single cells is revealing heterogeneity across different cells in transcript forms such as splice products and 5′ sequences [6–8, 12]. While substantial research has explored the molecular mechanisms of this variation [13–15], a key question remains: how does this transcriptomics variation map to external phenotypic variation? Is gene expression variation explained in part by cell physiological dynamics, such as metabolic phases of the cell like circadian rhythm or cell cycle [16]? Is the expression profile of a morphologically complex neuron more variable than that of a morphologically simpler cell, such as a brown adipocyte? Is there cell-type specificity or gene-class specificity to single-cell variability? To characterize the complexity and pattern of variation at the level of single cells we carried out single-cell RNA sequencing of multiple individual cells from five different mouse tissues, as well as rat samples for two of these tissues, with high depth of coverage. Most estimates of number of mRNA molecules in a mammalian cell suggest under ~300,000 molecules per cell [6]. With ~10,000 expressed genes, the average number of molecules per gene is ~30, suggesting that most of the transcriptome requires deep coverage and careful amplification for quantitative characterization. For this study, we used linear in vitro transcription for RNA amplification and quality controlled the RNA sequencing to include only those samples for which we had at least five million uniquely mapped exonic reads. Using this dataset as well as an extensive control dataset, we developed new analytical routines to carefully characterize patterns of gene expression variability at the single-cell level and dissected the cell-type-specific variability in relation to cell identity. We find evidence that single-cell transcriptome complexity and cell-to-cell variation have cell-type-specific characteristics and that patterns of gene expression variation may be subject to regulation.

## Results

### Single-cell RNA-sequencing datasets

**Fig. 1:**
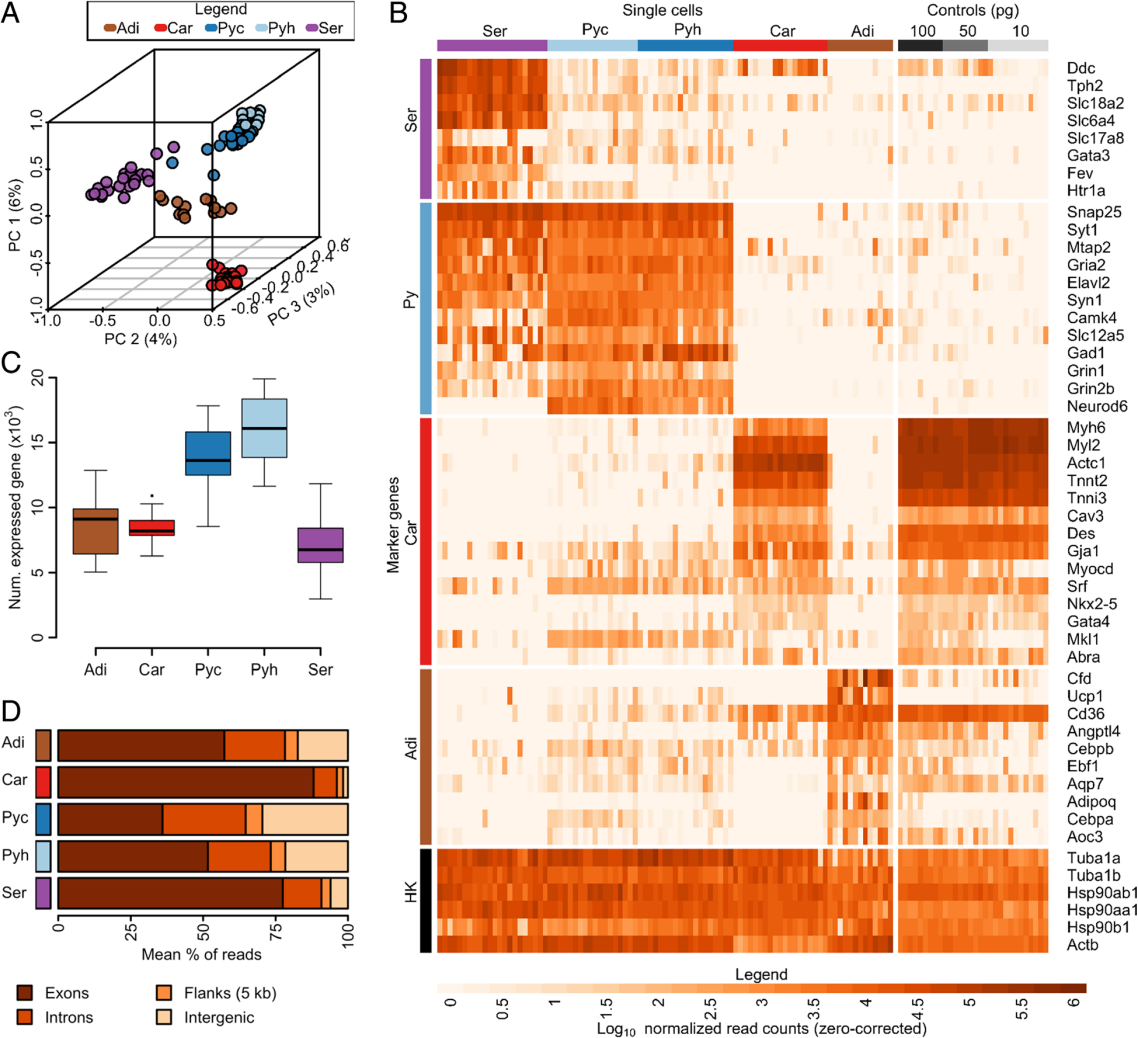
Single-cell dataset and transcriptome characteristics. **a** Low dimensional projection of single-cell transcriptome data. Axes were selected using principle component (*PC*) analysis of expression data. Relative frequencies of read counts were variance stabilized by arcsine transform. Genes with zero read count in all cells were excluded. Values in parentheses by each axis are percentage standard deviation explained by that axis. **b** Expression of marker genes (rows) selected from the literature [58–63] for all mouse samples (columns). **c** The number of expressed genes by cell type. **d** Average percentage of reads falling within annotated exons, introns, gene flanking regions and intergenic regions by cell type. Dilution control started with 100 pg (*100*), 50 pg (*50*), or 10 pg (*10*) total cardiomyocyte RNA. Abbreviations: *Adi* brown adipocyte; *Car* cardiomyocyte; *HK* housekeeping; *Py* pyramidal neuron; *Pyc* pyramidal neuron, cortex; *Pyh* pyramidal neuron, hippocampus; *Ser* serotonergic neuron, dorsal raphe

For each single-cell sample, we created a cDNA library after cell isolation that was linearly amplified by the antisense RNA (aRNA) method [17, 18] and then sequenced on the Illumina platform. From an initial 143 cells we identified 107 high quality samples with deep genic coverage, including 13 brown adipocytes, 19 cardiomyocytes, 19 cortical pyramidal neurons and 18 hippocampal pyramidal neurons from embryonic mouse, 8 cortical pyramidal neurons and 8 hippocampal pyramidal neurons from embryonic rat, and 22 serotonergic neurons from adult mouse (Tables S1 and S2 in Additional file 1). (Rat samples are included in cross-species comparisons, with primary analyses on mouse samples only. Unless otherwise specified, results are based on mouse data.) Several experimental parameters vary along with cell type, including age, collection method and culture conditions (as detailed in Materials and methods). In fact, since individual cells are the measurement units, all of our cell-type comparisons are confounded by the natural cell-specific phenotypes, such as lipid content and cell size. Such confounding is unavoidable at this level and interpretation of transcriptome characteristics should, therefore, all include this caveat. Nevertheless, each cell-type dataset is internally consistent (Table S1 in Additional file 1). In the resulting dataset, the average sample has a depth of 57 million reads with 17 million uniquely aligned to exons (minimum of 5 million unique exonic reads). Using these uniquely aligned reads, we assigned read counts to RefSeq annotated genes and normalized the dataset to mitigate differences in sequencing library depth [19]. Saturation curves generated by randomly subsampling reads for individual samples to generate synthetic replicates over a broad range of total depth demonstrate that little sensitivity is gained at increasing depth of coverage beyond five million unique exonic reads, suggesting sufficient depth (Figure S1a in Additional file 2). Additionally, within our range of coverage, we do not observe a relationship between the number of detected genes and the read depth (Figure S1b in Additional file 2). Principle components analysis projection of the 91 cells largely segregates the five cell types (Fig. 1a). We examined expression levels for a curated list of marker mRNAs expected in serotonergic neurons, pyramidal neurons, brown adipocytes and cardiomyocytes to validate the quality and identity of our samples (Fig. 1b). When clustered on these expression profiles the samples form coherent cell-type groups as expected, confirming dataset quality (Figure S1c in Additional file 2). While each cell type demonstrates a characteristic transcriptome profile enriched in expected marker mRNAs, we note that marker gene expression is rarely if ever limited to the expected cell type. As observed elsewhere, this suggests that multi-genic expression may better characterize cell types than expression of individual genes [20, 21]. In addition, some marker genes demonstrate substantial variability within the relevant cell type, suggesting that absolute expression levels of a small number of genes is not likely a critical determinant of cellular phenotype [21, 22]. We additionally prepared 30 control samples, amplifications of bulk total cardiomyocyte RNA diluted to single-cell quantities. All dilution replicates passed quality control thresholds and the set demonstrates high pairwise correlations (Tables S1 and S2 in Additional file 1; Figure S1d, e in Additional file 2). Compared with single cells, the dilution controls demonstrate generally larger pairwise correlations (Figure S1d in Additional file 2). For details on dataset preparation, see "Materials and methods" below.

### Single-cell transcriptome complexity

Averaged over all 91 cells, we observed 10,796 expressed genes per cell with 50 % of reads in a cell covering the 432 most highly expressed genes. The most abundant expressed gene comprises 2 % of reads on average, over 1000 times more than the median gene. Most expressed genes are observed in multiple cells, with a small fraction (0.027 %) of private genes expressed only in a single cell. We found pyramidal neurons (cortical and hippocampal cells) comprised a distinct transcriptome complexity group compared with the other three cell types. The number of expressed genes observed is significantly greater in pyramidal neurons (average of 14,964 genes) than in cells of the other three types (average of 7,939 genes, Welch’s t-test Bonferroni-corrected *p* < 0.05; Fig. 1c; Table S3 in Additional file 1). Significantly more genes were covered by 50 % of reads in pyramidal neurons compared with the other cell types and pyramidal neurons had higher numbers of private genes than the other cell types (Welch’s t-test Bonferroni-corrected *p* < 0.05; Table S3 in Additional file 1; Figure S1f, g in Additional file 2). Pyramidal neurons as a group displayed a larger fraction of reads mapping to non-exonic regions, especially the cortical cells, for which more than 60 % of reads mapped to introns and other non-coding sequences compared with a 42 % average for all cell types (Fig. 1d). The large portion of non-exonic sequences for all cell types is consistent with reports based on bulk data demonstrating that much of the genome is transcribed [23]. The larger percentage of non-coding sequences in pyramidal neurons is also in line with previous reports of long 3′ untranslated regions (UTRs) in the mammalian brain [24], with terminals well beyond annotated ends [25], and reports of intron retention in single neurons [7].

It is possible that difference in cell size and numbers of RNA molecules might affect detection sensitivity across cell types. While the cell sizes of the cell types used in this study have not be directly assessed, it is estimated that mammalian cells cover an eight-fold range in volume (BNID 100434 [26]). To adjust for this possible bias, we assumed an eight-fold difference in size between pyramidal neurons and other cell types and applied a corrected detection sensitivity threshold of eight times the minimum relative frequency observed in a given pyramidal sample, ignoring all genes below this threshold (validated on control data; Table S3 in Additional file 1; Figure S1h in Additional file 2). After correction all pairwise comparisons remain significant, with the exception of the brown adipocytes and cortical pyramidal neurons pair (Table S3 in Additional file 1; Figure S1h in Additional file 2).

Of the 371 genes found expressed in only one cell, 334 are in pyramidal neurons. These genes include 50 olfactory receptors genes, 10 vomeronasal receptors and 9 additional genes annotated with function in cell surface receptor-linked signaling, consistent with the hypothesis that these molecules create cell diversity within the central nervous system (Additional file 3; see Materials and methods for annotation sources). The presence of a large number of private genes, more detected genes, and greater non-coding expression all suggest unique transcriptional complexity in pyramidal neurons. While tissue studies have observed such complexity in the brain, here we identify this as a property of individual cells, not simply that of a highly diverse cellular population [24]. These data demonstrate that global transcriptome characteristics differ between cell types. We speculate that the broad transcription observed in single cortical and hippocampal neurons may be relevant for the phenotypic plasticity demonstrated by these cells, in contrast to the narrower functional repertoire required of heart and fat cells.

### Consistent gene expression across single cells

**Fig. 2:**
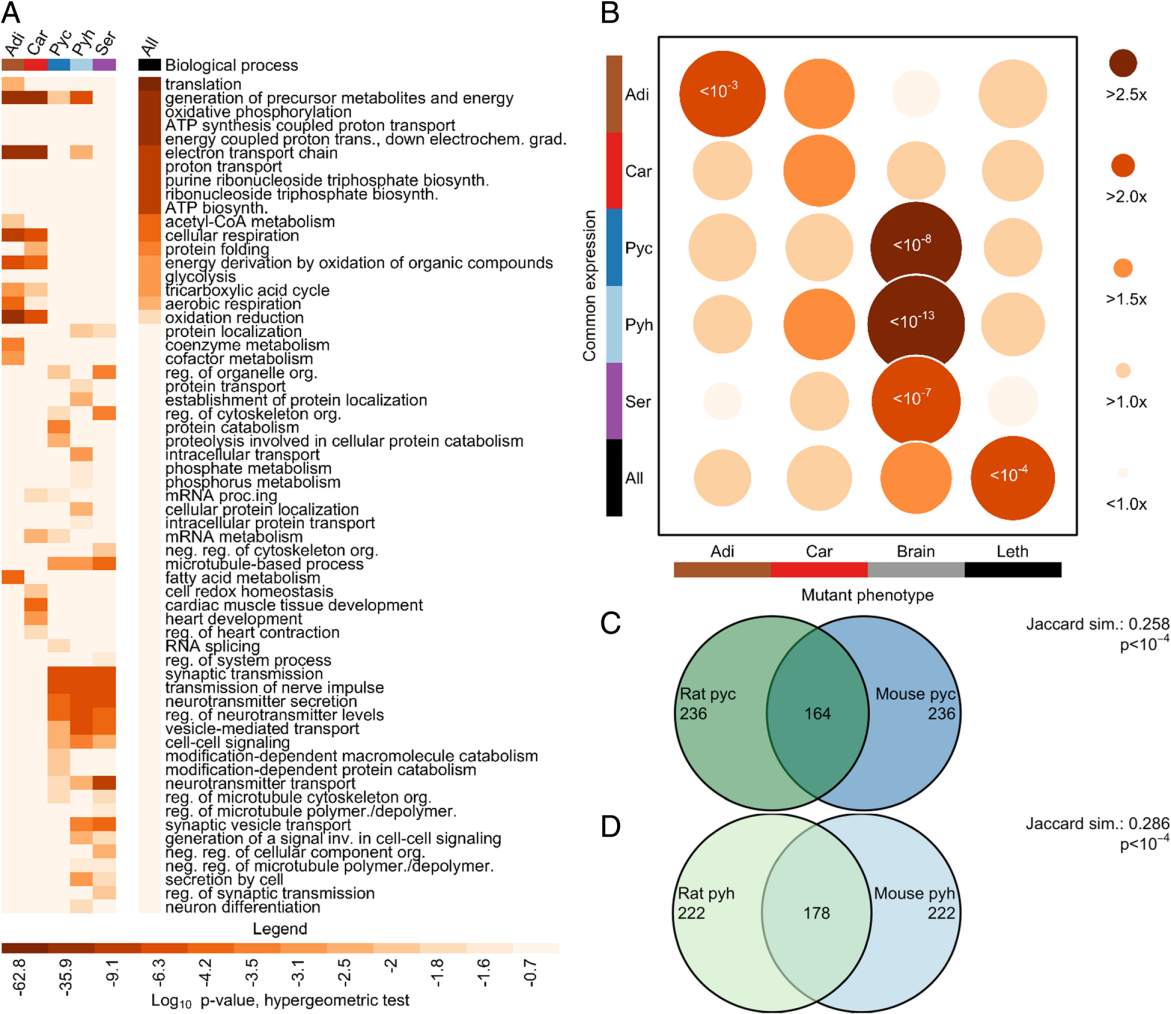
Phenotypic importance of genes with consistent expression. **a** Enriched Gene Ontology biological process categories across commonly expressed genes. Category abbreviations: *biosynth.* biosynthetic; *cmpd.* compound; *depolymer.* depolymerization; *deriv.* derivation; *dev.* development; *gen.* generation; *metab.* metabolites; *org.* organization; *oxid.* oxidation; *polymer.* polymerization; *prec.* precursor; *proc.* process; *reg.* regulation; *synth.* synthesis. **b** Association of common gene expression (rows) and mutant phenotypes (columns). Mammalian Phenotype Ontology phenotypes were grouped for affecting brown adipose tissue (*Adi*), myocardial tissue (*Car*), or brain tissue (*Brain*), or causing prenatal lethality [47, 48] (Table S7 in Additional file 1). Circle size indicates enrichment of phenotypic category in commonly expressed genes. Bonferonni-corrected *p* values are included for significant chi-square tests. **c, d** Overlap of common genes across species for cortical (c) and hippocampal (d) pyramidal neurons. *P* values were calculated by random sampling (see Materials and methods). Sample sizes and abbreviations: brown adipocyte (*n* = 13, *Adi*); cardiomyocyte (*n* = 19, *Car*); pyramidal neuron, cortex (mouse *n* = 19, rat *n* = 8, *Pyc*); pyramidal neuron, hippocampus (mouse *n* = 18, rat *n* = 8, *Pyh*); serotonergic neuron, dorsal raphe (*n* = 22, *Ser*)

Despite global transcriptional differences across cell types, we anticipated that all cells would constitutively express a subset of genes necessary for basic cell function. We identified 404 genes with evidence of expression in all 91 single cells (Additional file 3). Indeed, this set is enriched in functional annotations associated with housekeeping genes (hypergeometric test Bonferroni-corrected *p* < 0.05; Fig. 2a). We reasoned that if this gene set is critical for basic cell function, then gene disruption should be highly detrimental at an organismal level. We found that genes commonly expressed across all cell types are significantly more likely than remaining genes to be categorized as prenatal lethal, consistent with previous suggestions that genes whose deletion is lethal demonstrate low expression noise (Fig. 2b; Table S4 in Additional file 1) [13].

To examine commonly expressed genes within each of the five cell types, we selected the 400 highest expressed genes (defined by the minimum value in any cell) for each cell type, excluding the 404 universally expressed genes (Additional file 3). Because these gene subsets demonstrate common expression within cells of each type, we hypothesized phenotypic importance. As expected, this set of genes is enriched in annotations associated with cell-type-specific function, such as fatty acid metabolism, cardiac muscle tissue development, neuron differentiation and synaptic transmission (hypergeometric test Bonferroni-corrected *p* < 0.05; Fig. 2a). These genes are significantly more likely than expressed background to produce tissue-specific phenotypes on mutation but not to result in prenatal lethality (Fig. 2b; Table S4 in Additional file 1), with the exception of cardiomyocytes, which are proliferating and whose highly expressed genes are dominated by cell cycle function. The majority of commonly expressed genes, within each cell type and those that are universal in expression, do not have published phenotypic annotation and present a potential resource for disease association studies.

If common expression across single cells is indicative of critical gene function, this expression pattern may be conserved across species. We identified commonly expressed genes in rat cortical and hippocampal pyramidal neurons and compared them with the commonly expressed genes in mouse cortical and hippocampal pyramidal neurons (restricting analysis to unambiguous homologues and excluding homologues of universally expressed genes). The identities of cell-to-cell commonly expressed genes in each species show highly significant overlap (random sampling *p* < 10^−4^; Fig. 2c, d). That is, if a mouse gene tends to be commonly expressed in all pyramidal neurons (but not in every cell type), its rat homolog also tends to be commonly expressed, providing additional support that commonly expressed genes perform critical functions.

### Single-cell transcriptome variation

Because technical variability from single-cell RNA-sequencing measurements depends on their expression levels [27], we used the dilution replicates to determine a reliable range of gene expression before performing quantitative analyses (Figure S2a–d in Additional file 4). Examining replicates beginning with 10 picograms (pg) of total RNA (comparable to a single cell) [28], we identified an expression level that meets four reliability criteria: (1) at least 50 % of genes have no missing values in all dilution replicates (Figure S2a in Additional file 4); (2) variation across all single-cell samples is larger than twice that observed across dilution replicates (Figure S2 in Additional file 4); (3) variation across dilution replicates is approximately normally distributed (Figure S2c in Additional file 4); and (4) log read depth to log expression rank shows a consistent functional relationship (Figure S2d in Additional file 4). All criteria result in similar expression level thresholds: relative frequencies ranging from 2.6 × 10^−5^ to 6.3 × 10^−5^, corresponding to 328 to 789 reads. These conservative thresholds indicate reliable quantification of around four to nine input molecules. Approximately 25–37 % of the expressed transcriptome in a single cell is expected to have more than four to nine molecules (assuming 150,000 total mRNA molecules in a cell). For analyses below, we excluded genes with expression level below the most stringent threshold (relative frequency of 6.3 × 10^−5^). Note that use of thresholds based on replicates beginning with 10 pg of total RNA is conservative: thresholds selected for dilution replicates beginning with 50 or 100 pg of total RNA occur at lower expression levels (Figure S2a–d in Additional file 4).

**Fig. 3:**
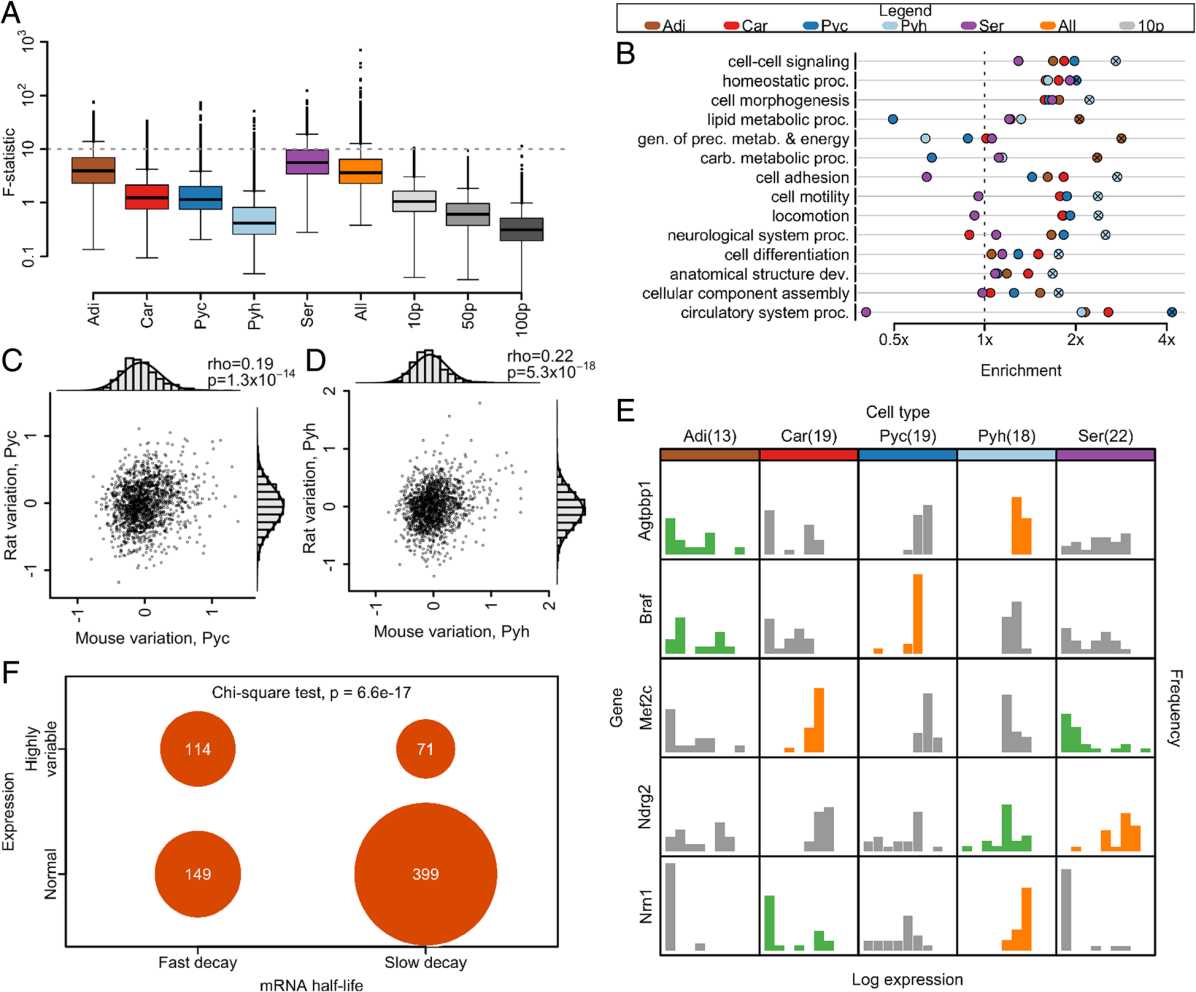
Cell-type patterns of transcriptome variability. **a** Distribution of expression variability across the transcriptome by cell type. **b** Enrichment of Gene Ontology categories among variable genes by cell type [49, 52]. *Crosses* indicate significance (Fishers exact test Bonferroni *p* < 0.05). Category abbreviations: *carb.* carbohydrate; *dev.* development; *gen.* generation; *metab.* metabolites; *prec.* precursor; *proc.* process. **c, d** Partial correlation of F-statistic across species, controlling for gene expression level, for cortical (c) and hippocampal (d) pyramidal neurons. Axes are a measure of variation, controlled for gene expression level (see Materials and methods for details). *rho* indicates the partial correlation coefficient. *P* values are from a two-sided t-test of association. Marginal histograms are shown overlaid with a normal curve. **e** Distribution of expression values by cell type for selected genes demonstrating highly variable expression by the outlier-sum statistic in one cell type and as following a normal distribution across cells in another cell type. Histograms of genes identified as highly variable in a given cell type are colored *green*; those of genes identified as normally expressed in a given cell type are colored *orange*. **f** Contingency table of gene categorization as hypervariable and as fast decaying [34]. Sample sizes and abbreviations: brown adipocyte (*n* = 13, *Adi*); cardiomyocyte (*n* = 19, *Car*); pyramidal neuron, cortex (mouse *n* = 19, rat *n* = 8, *Pyc*); pyramidal neuron, hippocampus (mouse *n* = 18, rat *n* = 8, *Pyh*); serotonergic neuron, dorsal raphe (*n* = 22, *Ser*); all single cells (*n* = 91, *All*); 10 pg dilution controls (*n* = 12, *10p*); 50 pg dilution controls (*n* = 9, *50p*); 100 pg dilution controls (*n* = 9, *100p*)

To characterize the extent of single-cell expression variation within each cell type, we calculated the ratio of biological variation over experimental variation observed at matched expression level (studentized F-statistic). This metric is a measure of total variation across single-cell samples (a combination of biological and experimental variation) relative to experimental variation. At a given expression level, larger values indicate larger biological variation. Briefly, we summarized the dependence of experimental variation (calculated across 10 pg dilution replicates) on expression level by computing a sliding window median (Figure S2e in Additional file 4). We scaled variation across single-cell samples by this sliding median value at matched expression level (Figure S3a–f in Additional file 5). As a negative control, we also calculated this statistic for dilution controls (Figure S3g–i in Additional file 5). For further details, see Materials and methods. We note that experimental variation (for the dilution controls, a combination of dilution and technical variation) depends on the gene-specific levels of RNA and the total amount of RNA (Figure S3g–i in Additional file 5; Fig. 3a). Differences in cell size could confound F-statistic distribution with total RNA effects. Because differences in total RNA molecules numbers for the different cell types are unknown, we use the F-statistic only to examine relative differences in gene expression variation within each cell type.

#### Within-cell-type variability

With the exception of hippocampal pyramidal neurons, all cell types demonstrate significantly greater transcriptome variability than that observed across 10 pg dilution controls (Wilcoxon rank-sum test *p* < 0.05; Fig. 3a). Every cell type contains highly variable genes with an F-statistic greater than 10 (Fig. 3a), indicating the presence of highly variable genes for each cell type. To compare the extent of transcriptome variation across cells of the same type with transcriptome differences across cell types, we computed the variance within each cell type as well as the variance between each pair of cell types (Table S5 in Additional file 1). We found that expression variation within some of the cell types is comparable to that observed between some of the cell types: within cells of the same type, variance ranges from 0.21 to 1.31, while between cell types variance ranges from 0.26 to 1.88. This result may be affected by the difference in total RNA content of each cell. Nevertheless, this suggests that a great diversity of transcriptome states may support an equivalent external cell phenotype.

To assess whether variability might be related to cell-type-specific sub-states, we identified the 5 % most variable genes in each cell type by the F-statistic (Additional file 3; Figure S3a–i in Additional file 5). We tested these genes for enrichment of Gene Ontology molecular function and biological process categories (Fig. 3b; Table S6 in Additional file 1). Functional categories relevant for plastic phenotypes are enriched among variable genes in a cell-type-specific manner. Functional categories are enriched among variable genes in a cell-type-specific manner. In pyramidal neurons, variable genes are enriched in functions important to cell migration, such as cell morphogenesis and locomotion. Generation of precursor metabolites and energy is enriched among variable genes in brown adipocytes. But we note similar enrichment is also seen among 50 pg and 100 pg dilution controls, which may be due to the effect of expression level on the F-statistic (Table S6 in Additional file 1). This suggests that, for a subset of genes, the observed variability is due to cell-type-specific molecular physiology.

We also examined the degree of expression variation among different functional classes of genes by calculating the F-statistic for several broad categories (Table 1). Genes categorized with classic housekeeping functions (mitochondrial or ribosomal function) demonstrate low expression variability, while transcription factors, important in responding to the environment or modulating cell function over time, demonstrate significantly greater gene expression variability. Interestingly, ion channels demonstrate the largest variability, significantly larger than all other examined categories, possibly suggesting homeostatic modulation [29, 30].

**Table 1:** Gene expression variability of different functional gene categories

Gene category 1	Gene category 2	Difference in adjusted mean expression variability (log_10_ F-statistic)		
		Category 1 – category 2	95 % CI	*p* value
Ion channel	Metabolism	0.32	0.22–0.41	<10^−5^
Ion channel	Ribosome	0.33	0.23–0.42	<10^−5^
Ion channel	Transcription factor	0.16	0.07–0.25	4.84 × 10^−5^
Transcription factor	Metabolism	0.16	0.11–0.20	<10^−5^
Transcription factor	Ribosome	0.17	0.12–0.22	<10^−5^
Ribosome	Metabolism	−0.01	−0.06–0.04	0.92

Comparison of gene expression variability across functional gene categories controlling for gene expression level. Adjusted mean values were calculated using a two-factor ANCOVA of log_10_ F-statistic, with functional gene category and cell type as independent factors and conditioning on gene expression level (log_10_ average normalized read depth). Adjusted means are reported at the average gene expression level. Reported *p* values are for a two-sided Tukey’s test and are calculated based on a joint t-distribution to control the family-wise error rate

If gene expression variation amongst individual cells is important for tissue function, the degree of variation itself may be conserved across species. We calculated the F-statistic for cortical and hippocampal pyramidal neurons in rat, filtering genes by the quality control threshold described above. For each cell type we computed the partial correlation of the F-statistic across species, controlling for gene expression levels to ensure that correlation was not simply due to shared levels of gene expression (see "Materials and methods" for details). The partial correlation coefficient across species is significant for both cell types examined (two-sided t-test of association *p* < 10^−13^; Fig. 3c–d). The number of cells from rat is relatively small and further studies are required to confirm that gene expression variation is conserved across species. Furthermore, additional data are needed to dissect whether such conservation in variance is cell-type-specific or indicative of more general selection for tight regulation of those genes that are critical for global cell function. Lastly, as in our other results, any statement on variances must be interpreted cautiously because of the intensity-dependence of variance and analytic techniques for variance stabilization. But the current comparative data are consistent with the hypotheses that gene expression variation is regulated, at least for some genes, and that the pattern of gene expression across a population of cells might be important for tissue function.

#### Patterns of expression variation

We next examined a subset of genes with patterns of extreme variability across cells of the same type. To identify these genes, we used the outlier-sum statistic, a method proposed to detect genes with high expression in a subset of sample (Additional file 3) [31, 32]. As controls, we also identified variable genes across all cell types (a positive control) and across dilution replicates (a negative control) by the same method. Genes identified across all cell types are enriched for categories relative to cell-type-specific functions, including heart development, behavior, and regulation of system process, a category encompassing processes that modulate tissue function (Table S6 in Additional file 1). No functional categories are enriched among genes identified across dilution replicates. Genes identified across cardiomyocytes, which were collected from embryonic tissue undergoing cell division, are enriched for function in mitosis, nuclear division and organelle fission; and genes identified in brown adipocytes, which generate heat and fever and share some immune regulators, are enriched in immune response related genes. While only a small number of genes were identified in pyramidal neurons, limiting the ability to detect functional enrichment, individual genes demonstrate tissue-specific function. The most highly ranked genes in cortical neurons include *Crh*, with function in associative learning, long-term memory and response to cocaine; *Vip*, again with function in learning and memory; and *Tac2*, involved in associative learning and long-term memory formation in humans (Additional file 3). The functional coherence among these genes again raises the possibility that single-cell variation, at least in a subset of genes, is regulated.

We identified 58 genes demonstrating qualitatively different expression patterns in different cell types. Each of these genes is classified as highly variable by the outlier-sum statistic in at least one cell type, and as following a normal distribution across cells in at least one other cell type (Fig. 3e; Additional file 3). Each gene is highly expressed in multiple cell types (due to use of quality control threshold), within the top 36 % of expressed genes, but demonstrates a markedly different expression distribution in different cell types. This difference of expression pattern of the same gene in different cell types also suggests that this expression variation may be controllable and the result of regulation.

For this set of 58 genes we do not find significant enrichment for any Gene Ontology classification. But we observed individual cases of genes of note that are associated with cell-type-specific function. For example, mutants of *Nrn1*, a gene following a normal distribution across hippocampal neurons, are associated with abnormal spatial learning and impaired contextual conditioning behavior. Mutations of *Braf*, a gene following a normal distribution across pyramidal neurons, are associated with abnormal learning and abnormal hippocampal granular and cerebral cortex pyramidal neuron morphology. *Mef2c* follows a normal distribution across cardiomyocytes; this gene functions in cardiac muscle development and has been used in transdifferentiation experiments converting fibroblasts to cardiomyocytes [33]. These examples again suggest that consistency in gene expression may be an indicator of critical phenotypic relevance.

#### Association of extreme variability with RNA half-life

Because variation in gene expression may be buffered by long RNA half-life, we hypothesized that if extreme variability is generated by transcriptional switching, these genes may demonstrate rapid decay. We categorized genes as having slow or rapid decay based on publically available RNA half-life measurements and then tested for an association with variability [34]. Genes identified as variable are significantly more likely to be classified as rapidly decaying than genes with highly consistent expression (Chi-square test *p* < 10^−16^; Fig. 3f). While a rapid decay does not necessarily indicate large expression variability, highly variable genes rarely have slow decay and genes with slow decay are rarely highly variable (Figure S3j in Additional file 5). This is consistent with models of transcription, which suggest that bursts of changes in RNA numbers may occur when the intervals of inactive transcription are long relative to mRNA decay [15]. A rapid decay rate of a transcript may enable rapid changes in gene expression levels.

### Sequence variation at the single-cell level

**Fig. 4:**
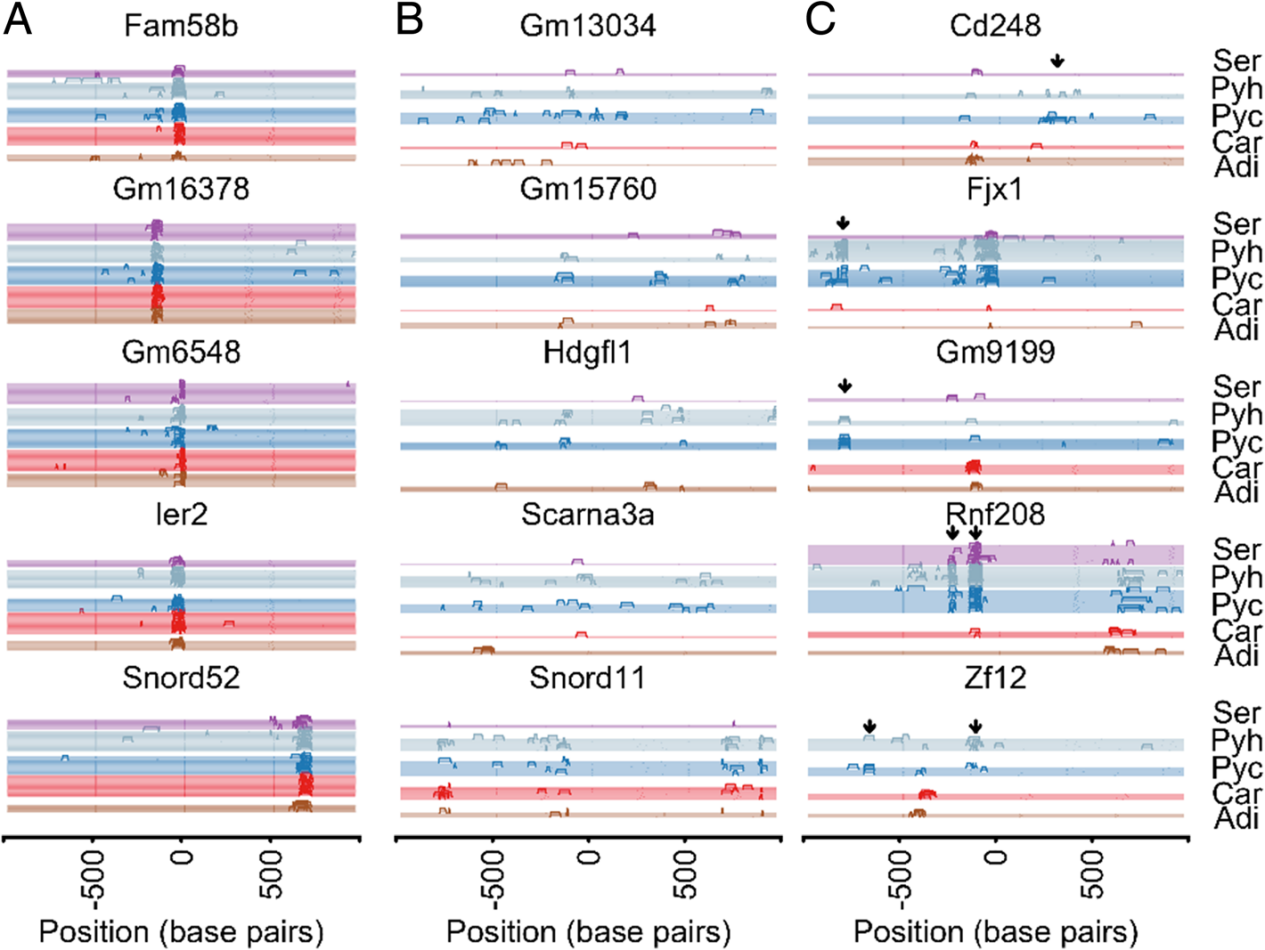
3′ UTR usage across single cells. Each row represents a single cell and line height indicates the presence of a 3′ UTR termination site. The x-axis ranges from 1000 bases upstream to 1000 bases downstream of the annotated end site with the annotated end site at position zero. Empty rows indicate no detected expression or 3′ UTR termination site within the examined 2000-base-pair region. **a** Highly consistent 3′ UTR termination sites across cell types. **b** Highly variable 3′ UTR termination sites across all cells. **c** 3′ UTR termination sites with cell-type-specific usage. Abbreviations: *Adi* brown adipocyte; *Car* cardiomyocyte; *Pyc* pyramidal neuron, cortex; *Pyh* pyramidal neuron, hippocampus; *Ser* serotonergic neuron, dorsal raphe

The nucleotide resolution of RNA sequencing allows detection of transcript isoform expression. To examine isoform usage across our single-cell dataset, we took advantage of characteristic strand alignment patterns that result from Illumina sequencing chemistry at the ends of transcripts. Because aRNA amplification allows highest coverage at the 3′ end of transcripts, we focused our attention on identification of 3′ UTR terminals in single cells. In addition, we restricted our attention to single-exon genes to ensure high confidence in observed isoform usage patterns. In 132 genes, we observe highly consistent usage of a single 3′ UTR poly(A) or termination site across cells of all types, though the observed site may not agree with the annotated end location (Fig. 4a). These genes are enriched in transcription regulation, including transcription factors like *Fam58b*. A second set of 163 genes demonstrates highly variable 3′ UTR termination sites across all cells and cell types (Fig. 4b). Most intriguingly, 74 genes demonstrate cell-type-specific usage of 3′ UTR termination sites (Fig. 4c). For example, pyramidal neurons use a short isoform of both *Gm9199* and *Fjx1* which are different in other cell types. While the function of *Gm9199* is unknown, knockout of *Fjx1* results in abnormal dendrite morphology. All pyramidal neurons demonstrate expression of both the canonical end site of *Cd248*, in addition to a longer isoform observed rarely in other cell types. This gene encodes a protein integral to the membrane that functions in cell migration and the unique isoform usage in pyramidal neurons suggests a potentially interesting neuronal role. Because differential 3′ UTR usage often facilitates differential mRNA regulation — for example, through control of translation or localization — the occurrence of different isoforms across cell types suggests cell-type-specific post-transcriptional regulatory dynamics.

## Discussion

Cell-type-specific characteristics of the single-cell transcriptome recapitulate characteristics of tissue-level expression data, indicating that, at a minimum, single-cell RNA sequencing is meaningful at the level of cell-type identity. In addition, our data suggest that expression variation among individual cells of the same type has biological significance. Fig. 1a, a principle components analysis projection of the 91 mouse cells, displays the overall pattern of transcriptome variation and suggests a complex pattern of single-cell variability both within each of the five cell types and between them. The analyses described above suggest that these expression patterns may be driven by a multitude of factors. The enrichment of certain functional classes among variable genes suggests that some observed variability is due to measuring cells in different phases of functional dynamics. The greater transcriptome complexity we see in pyramidal neurons, both in the number of expressed genes and in the degree of non-coding expression, suggests that the complexity of available RNA may be related to the morphological complexity and plasticity of a cell. Enrichment of cell surface receptor molecules among private genes, as well as the occurrence of variable 3′ UTR usage, suggests that some variation at the level of individual cells might be “programmed” to induce a heterogeneous assembly of cells in a tissue, perhaps to carry out organ level function. Some of the variation is also likely to be due to molecular noise from a finite number of molecules [15]. However, we also see a high degree of variation for highly expressed genes and the multi-dimensional pattern of variation suggests a large degree of within-cell-type dispersal.

At the level of current measurement technology, observed cell-to-cell variation will be confounded by technical variability. It is also possible that individual cells' phenotypes, such as size and ultrastructure, may affect cell-to-cell transcriptome variability. However, such single-cell phenotype variation may also be a biological rationale for transcriptome variability. We cannot rule out that observed within-cell-type variation is due to technical difficulties but multiple signatures from our analyses suggest that, at least for a distinct subset of genes, there is significant biological variation even for cells that are nominally identified with the same cell type.

Single-cell transcriptome measurements are becoming increasingly common with a variety of techniques for RNA amplification and sequencing. In particular, we emphasize the need for careful control data as well as statistical analysis routines that incorporate the unique properties of single-cell transcriptomes. Another key aspect of inference from single-cell data is the depth of read coverage required to quantitatively characterize most of the transcriptome. Using statistical analysis of the control data and other characteristics of our data we estimate that with the aRNA technique we can conservatively measure with quantitative precision four to nine molecules of input mRNA, which is approximately 25–37 % of the genes that are expressed in a single cell. A difficult problem is that measurement variation is likely a function of absolute numbers of molecules. Individual cell characteristics, such as the number of RNA molecules, size of the cell, and cell components that interact with RNA recovery, will all confound technical variability. Overall statistical characteristics of within- and between-cell-transcriptome variations are expected to be consistent and informative. But any given measure will include interactions of both biological and technical factors. This is a problem that will be endemic to any single-cell quantitative measurements.

## Conclusions

Summarizing our data, the functional coherence of genes identified based on expression variation, tissue-level phenotypes in animals with mutational knock-outs of consistently expressed genes, and correlation of expression patterns across rat and mouse leads us to hypothesize that some observed variation is necessary for tissue-level function of the cell and that the degree of single-cell variation in gene expression may be under regulatory control. In addition, we hypothesize that the same external cell phenotype may be robustly produced from a great diversity of transcriptome states, as long as these states remain within some bounds. Gene expression is required to maintain the multi-genic stoichiometric constraints of a cell’s normal physiology but such constraints may allow many degrees of multi-dimensional freedom [35]. Thus, we propose there are both functional and degrees-of-freedom rationales for the high degree of single-cell transcriptome variation. Similar determinants of variation are found in many biological systems comprised of aggregates of functional units and we suggest that cells within organs may be more like individuals in an ecological community rather than homogeneous replicate units.

## Materials and methods

### Cell culture and single cell isolation

Primary cultures of embryonic day 18 (E18) hippocampal and cortical neurons from mouse (C57BL/6, Charles River Laboratories, Inc.) and rat (Sprague Dawley) were cultured as previously described [36]. Interscapular brown adipose tissue was extracted from E17.5 CD-1 mice and cultured for one day as described elsewhere (Spaethling JM, Sanchez-Alavez M, Lee J, Xia FC, Dueck H, Wang W, Fisher S, Sul JY, Seale P, Kim J, Bartfai T, Eberwine J: Single-cell transcriptomics and functional target validation of brown adipocytes show their complex roles in metabolic homeostasis, submitted). We identified pyramidal neurons and brown adipocytes by cell morphology, isolated single cells with patch pipettes, deposited collected material into an eppendorf tube and froze it immediately at −20 °C until storage at −80 °C [17]. Serotonergic neurons, identified with yellow fluorescent protein (YFP) expression, were isolated by pipette directly from acute slices of the dorsal raphe of P60 ePet-YFP mice as described elsewhere [37, 38]. Ventricular cardiomyocytes were isolated from E14.5 transgenic mice expressing the green S/G2/M fluorescent ubiquitination-based cell cycle indicator “Fucci” [39] using a modified protocol of the neomyts cardiomyocyte isolation kit (Cellutron Life Technologies). The Fucci probe, monomeric Azami Green (mAG) fused to the ubiquitylation domain of human Geminin [*hGem* (1/110)] highlights the green nuclei of individual cycling cardiomyocytes in the S/G2/M phases of the cell cycle. Dissociated cell suspensions were resuspended in 500 μl of phosphate-buffered saline supplemented with 5 % fetal bovine serum solution and sorted by flow cytometry on a FACSAria instrument operating at low pressure (20 psi) using a 100-μm nozzle. Cardiac cells expressing mAG-*hGem* (1/110) transgene (mAG positive) were identified using a sequential gating strategy for size, doublet discrimination, viability staining with 7-aminoactinomycin D (7AAD) and mAG fluorescence intensity using the FACSAria 488-nm excitation laser. FACSDiva software was used for data acquisition and analysis. The mAG-positive and mAG-negative single cells were sorted individually into 96-well plates containing reverse transcriptase buffer for linear amplification as described below. Following the first strand cDNA, the molecular identity of the sorted cardiomyocytes was confirmed by PCR for positive expression of the cardiomyocyte-specific gene *Tnnt2* and negative expression of *Pdgfrb* (platelet derived growth factor receptor, beta polypeptide) to exclude potential contamination by other cardiac cell types, such as cardiac fibroblasts and endothelial cells.

Collection of the primary cultured cells utilized an animal byproducts protocol, “Genome Biology of Single Neuron Function and its Modulation with Age” under University of Pennsylvania IACUC protocol #803321 (approval 22 September 2010). Animals were sacrificed under University of Pennsylvania IACUC protocol #804867, “Molecular Biology of Single Aging Neurons and Glia” (renewal 15 May 2013), but sacrifice of the animals was independent of the work reported in this paper. The cardiomyocytes were collected under Harvard University IACUC protocol 12-05-2169R, “Mechanisms of myocardial regeneration” (approval 23 May 2012). All protocols were approved by University of Pennsylvania and Harvard Office of Regulatory Affairs and IACUC committee. After 2013, the PENN IACUC classified byproduct protocols for cells as procedures that do not require prior regulatory approval.

### mRNA amplification and library construction

Collected samples were individually amplified using three rounds of a linear in vitro transcription-based method described elsewhere [17]. Amplified material was quantified and size-checked using a Bioanalyzer RNA Nanochip (Agilent), then prepared for multiplexed paired-end sequencing using the TruSeq or mRNA-Seq systems according to the manufacturers' instructions. Initial mRNA selection steps were skipped to accommodate aRNA amplified material. Samples were sequenced on HiSeq instruments to produce 100-base paired-end reads. Sample-specific sequencing data can be found in Table S2 in Additional file 1.

### Dilution controls

To control for technical variation arising during amplification and sequencing preparation, we performed replicate amplifications of bulk RNA diluted to near single-cell quantities. The starting bulk sample was heart tissue total RNA from a C57/BL6 adult male mouse (Zyagen). Twelve amplifications were begun with 10 pg of total RNA, nine with 50 pg and nine with 100 pg. After three rounds of aRNA amplification quality and quantity of all samples were assessed using Nanodrop and Bioanalyzer RNA Nanochip. Samples were then prepared for sequencing using the stranded TruSeq mRNA protocol (Illumina). Replicates were sequenced as above.

### Alignment, quantification and sample selection

For mouse samples, we trimmed reads for adapter and poly(A) contamination using in-house software before aligning to the mouse genome and transcriptome using RNA-Seq Unified Mapper (versions 2.0.2_06 and 2.0.3_04) and mouse genome build mm9 [40]. Uniquely aligning reads with three or fewer mismatches per 100 bases were retained for further analysis. Using RefSeq annotations downloaded from the UCSC genome browser in December, 2012, we assigned read counts to genes using HTSeq and htseq-count [41, 42]. Only exonic reads were counted, with overlap assigned using the intersection non-empty method. Note that the use of uniquely aligned exonic reads means that genes with limited unique sequence will be largely missed in this analysis. Though this is rare, one notable example is the housekeeping gene *Gapdh*. Rat samples were aligned with STAR [43] to rat genome build rn5. Processing after alignment was completed as described above with rn5 RefSeq annotations for gene quantification (downloaded from the UCSC genome browser).

We further filtered samples to retain those with high genic read coverage (greater than five million uniquely mapping exonic reads) and to create an overall dataset with similar sequencing library characteristics. Every included mouse sample was required to have at least two out of the three following traits: at least 50 % uniquely mapping reads, at least 1500 genes covered at reasonable depth (greater than ten reads), and less than 80 % short fragments (inferred from the percentage of uniquely mapping read pairs where the mate pair alignments overlap). Because the statistic used to identify short fragments is distinctive to RUM alignment reports, we report no comparable statistic for the STAR-aligned rat samples. For these samples, we required at least one of the two remaining criteria. Quality information for each sample can be seen in Table S2 in Additional file 1.

To mitigate differences in read counts due to variable sequencing depth, we normalized the resulting dataset by the method proposed by Anders and Huber [19]. In this method, a pseudo-reference sample is generated by taking the geometric mean across samples of ubiquitously expressed genes. A size factor estimating the contribution of sequencing depth is estimated as the median ratio of expression of ubiquitously expressed genes in a single sample to the pseudo-reference. All read counts for that sample are then adjusted by this factor. All further analyses, except those using relative frequencies, used normalized read counts.

Because both GC content and gene length have been shown to affect RNA-sequencing measurements, we tested for a relationship between these gene traits and normalized gene counts (Figure S2f in Additional file 4) [44]. We tabulated gene length and GC content for mouse RefSeq genes, with the exception of 43 genes with annotated positions on multiple chromosomes. Correlations of these traits with gene counts are negligible.

We performed principle component analysis of gene expression data after variance stabilizing relative frequencies by arcsine transform. Genes with zero read count in all cells were excluded. To visualize a projection of the data on the three components with largest singular values, we used the R ‘scatterplot3d’ library [45].

### Characterization of single-cell transcriptomes

All statistics were computed in R [46]. t-Tests used to test the null hypothesis of no difference in means were performed for groups with different sample sizes and different variance, with rejection of the null hypothesis at a Bonferroni-corrected *p* value of 0.05. Expressed genes include any gene with at least one uniquely aligned read. Private genes include any gene with at least one uniquely aligned read in a single cell, but none in any other cell. The functional and phenotypic annotations for gene sets throughout the paper were found via Mouse Genome Informatics or DAVID Functional Annotation Tool, using Gene Ontology molecular function and biological process classification, as well as Mammalian Phenotype Ontology classification [47–51]. Mammalian Phenotype Ontology annotations were accessed through Mouse Genome Informatics on June 2013, excluding annotations based exclusively on cell line experiments [47, 48]. Gene Ontology annotations for all expressed genes were also downloaded June 2013 from Amigo [52].

To characterize cell-type patterns of transcription genome-wide, we first classified regions of the autosomal genome as exonic, intronic, flanking or intergenic. To do this, we accessed the following annotations for the mm9 reference genome from the UCSC genome browser in March 2013: miRBase, RefSeq genes, Ensembl Genes, Vega genes and UCSC known genes [41, 53]. Any region annotated as an exon for a gene or non-coding RNA from any of these sources was classified as exonic. Regions internal to transcribed units but not annotated as exonic by any annotation were classified as intronic. Regions 5 kilobase pairs upstream and downstream of any transcribed unit, or up to the nearest neighboring exonic region, were categorized as flanking regions. All remaining genomic regions were categorized as intergenic. Reads were assigned to these regions using HTSeq via the intersection non-empty method as above. For each sample, the fraction of reads assigned to each region was calculated and the mean value for each cell type is shown.

### Consistent genes

Genes with universal expression were defined as genes with at least a single read in all samples. Enriched functional terms were found using DAVID Functional Annotation Tool to be significant relative to *Mus musculus* background by hypergeometric test at a Bonferroni-corrected *p* value of 0.05, using Gene Ontology biological process FAT annotations [49–51]. In cases where the identical gene subset was enriched in multiple terms, the most significantly enriched term(s) was (were) reported. As for universally expressed genes, commonly expressed genes were found for each cell type. Of all commonly expressed genes within a particular cell type, the top 400 genes were selected by the minimum read count in any cell excluding universally expressed genes. Functional enrichment for these gene sets was found as above. There were 3752 expressed genes with Mammalian Phenotype Ontology annotations and these were used to test the association between common expression and prenatal lethality and between common expression and tissue-specific mutant phenotypes. For annotations assigned to these phenotypic categories, see Table S7 in Additional file 1. Because a very small number of expressed genes had phenotypic annotations exclusive to a single brain tissue, we excluded brain tissue-specific terms and instead focused on terms with broader neuron or brain phenotypes. Association was tested using chi-square tests, rejecting the null hypothesis of no association at a Bonferroni-corrected *p* value of 0.05. Enrichment was calculated as the fraction of common genes with phenotype relative to the same fraction for remaining genes.

To compare common genes across species, we downloaded homologue annotations from Mouse Genome Informatics in February 2015 [47, 48] and filtered homologues to include only unambiguous cases with one assigned gene in each species. Common genes were identified in each cell type as described above separately for each species, excluding homologues of mouse universally expressed genes. For each cell type, we calculated the Jaccard index of identified common genes as a measure of gene list similarity. To determine whether the observed similarity was significant we randomly sampled gene lists of matched size from each species, computing the Jaccard index for each. The assigned *p* value is the fraction of observed similarities of the same or greater value than the true index in 10,000 random samples.

### Gene expression variability

Our aim was to identify biological variation across single cells and limit the effect of technical variation on our conclusions. Because single-cell RNA-sequencing experiments are subject to technical variation dependent on expression level [27, 28], we generated an estimate of experimental variation as a function of expression level to use as a baseline measure of technical variation. We generated this control curve using a kernel approach, first computing variation across twelve 10-pg dilution replicates for all genes, then summarizing the variation at a given expression level as the median value across 500 neighboring genes (Figure S2e in Additional file 4). We then used an F-statistic as our measure of variation across single-cell samples, scaling observed variation by control variation at matched expression level. Specifically, for gene g_i_ with average expression level x_i_, we calculate:

$$ {\mathrm{F}}_{\mathrm{i}} = {\mathrm{V}}_{\mathrm{total}}\left({\mathrm{g}}_{\mathrm{i}},\;{\mathrm{X}}_{\mathrm{i}}\right)/{\mathrm{V}}_{\exp}\;\left({\mathrm{X}}_{\mathrm{i}}\right) $$

where V_total_ is the total sample variance calculated on relative frequencies for a given cell type, and V_exp_ is an estimate of experimental variation as a function of expression level, as described above. Because the total observed variance is a combination of biological and technical variation, larger values for this measure at a given expression level indicate larger biological variation. As an additional negative control, we also computed the F-statistic for the dilution replicates against the control curve and include this group in variation analyses.

The F-statistic is sensitive to non-normality, but it is not generally true that experimental variation in single-cell RNA sequencing is normal, particularly for genes observed only in a subset of replicates. We expected that there may be an expression level beyond which missing data rarely occurs and experimental variation is approximately normal. To identify such a threshold, we computed the Shapiro-Wilk statistic separately for all genes observed in 10-pg dilution replicates. As for the variation control curve, we found the median curve for Shapiro-Wilk *p* values and identified the expression level beyond which all median *p* values are greater than 0.01 across dilution replicates (Figure S2g in Additional file 4). The selected threshold (relative frequency of 6.32 × 10^−5^) corresponds approximately to a read depth threshold of greater than 789 reads, resulting in retention of less than 25 % of genes for each cell type. Genes with average relative frequencies below this threshold for a given cell type were excluded from variation analysis. We additionally identified expression level thresholds that satisfy three further quality-control criteria (see "Results"; Figure S2a–d in Additional file 4). All produce similar, though slightly lower, expression level thresholds. We excluded genes with mean expression below the most stringent threshold from all variation analysis. We note that thresholds identified based on dilution controls beginning with larger amounts of input RNA occur at lower expression levels and are less stringent than the threshold used.

As for gene counts, described above, because both GC content and gene length have been shown to affect RNA-sequencing measurements, we checked for a relationship between these gene traits and the above-described F-statistic and found that correlations between the traits and measure are negligible (Figure S2h in Additional file 4). We additionally examined the relationship of the F-statistic with expression level (Figure S3a–i in Additional file 5). The measure is not strongly dependent on expression level, though for biological samples the largest F-statistic values occur at the highest gene expression levels. This is appropriate, since biological signal will be most clearly distinguishable from experimental variation at high expression levels; however, genes ranking within the top 5 % by F-statistic value above the expression level threshold are generally found to span a broad range of expression levels (Figure S3a–f in Additional file 5).

To test whether functional gene categories demonstrate different degrees of expression variability, we compared mean F-statistic values across genes categorized as metabolic, ribosomal, transcription factor, or ion channel. Annotations for ion channels were accessed from the International Union of Basic and Clinical Pharmacology public database (downloaded June 2013) [54], and for transcription factor activity, ribosomal function, or metabolic function from Gene Ontology. Because the F-statistic depends on expression level (see above) and because different functional gene categories may have different mean expression levels, we desired to compare expression variability across categories while controlling for gene expression level. To do this, we performed a two-factor ANCOVA of log_10_ F-statistic with functional gene category and cell type as independent factors and conditioning on gene expression level (log_10_ average normalized read depth). Cell type was included as a cofactor to control for global differences in F-statistic distributions across cell types. Log transformations were used to satisfy normality assumptions. Adjusted means for each gene category were found at average covariate values. Post hoc comparison of adjusted means was performed using Tukey’s test and *p* values were calculated based on a multivariate t-distribution to control family-wise error rate. Several R packages (car, effects and multcomp) were used to perform this analysis [55–57].

To compare expression variability across species, we filtered genes to retain those with unambiguous (single) homologues in both rat and mouse, as described above. Additionally, only genes passing the quality control expression level threshold in both species were considered. Because the F-statistic depends on expression level (see above), we wished to control for expression level in measuring expression variability similarity because we anticipate that expression level may be conserved. For this reason, we used partial correlation as a measure of similarity across species, conditioning on the mean expression level for each species. Specifically, for each species we fit the model:

$$ \mathrm{l}\mathrm{o}{\mathrm{g}}_{10}\left(\mathrm{F}\hbox{-} \mathrm{statistic}\right)={\mathrm{b}}_0+{\mathrm{b}}_1\mathrm{l}\mathrm{o}{\mathrm{g}}_{10}\left({\mathrm{X}}_{\mathrm{mouse}}\right)+{\mathrm{b}}_2\mathrm{l}\mathrm{o}{\mathrm{g}}_{10}\left({\mathrm{X}}_{\mathrm{rat}}\right) $$

where x_mouse_ and x_rat_ refer to mean expression values. Partial correlations were calculated using residuals. Significance was calculated using a two-sided t-test for association, rejecting the null hypothesis of no association at *p* < 0.05.

To identify genes with patterns of extreme variation within each cell type, we employed the outlier-sum statistic [31, 32]. Briefly, this statistic is a measure of the presence of extreme outliers. Genes are median-centered and scaled by the median absolute dispersion. To avoid filtering genes with outliers, if a gene has zero median absolute dispersion, it is instead scaled by the minimum observed non-zero value across the transcriptome. Statistical outliers are identified and their standardized values are summed. Genes classified as variable ranked among the top 400 by this statistic in a given cell type and additionally had an outlier-sum of at least 100. Consistently expressed genes ranked among the top 400 in a given cell type by the Shapiro-Wilk statistic, which measures similarity to a normal distribution, and additionally had a probability of normality greater than 0.005. Functionally enriched categories were identified against matched background sets of expressed genes.

To test the hypothesis that genes with patterns of extreme variation have short RNA half-lives, we used publically available RNA half-life measurements [34]. We classified gene stability following the method used by the original authors, ranking genes by half-life and categorizing the upper third as slow decaying and the lower third as fast decaying. We tested for an association between categorization of genes as highly variable or consistently expressed and as rapidly or slowly decaying using the chi-square test, rejecting the null hypothesis of no association at *p* < 0.05.

### Detection of 5′ and 3′ ends of transcripts using Illumina paired-end sequencing

Standard Illumina paired-end RNA library preparation involves fragmenting the RNA template, attaching adaptors, creating double-stranded cDNA, amplifying the products, and reading the sequences from each end of the cDNA. Assume that the RNA fragments have been approximately size selected and that we carried out *k* base pair-length paired-end sequencing. Suppose the fragmented RNA template is represented as 5′-PQR-′3, where P represents *k* base pairs of the sequence at the 5′ end, R represents *k* base pairs of sequence at the 3′ end, and Q represents *s* base pairs of sequence in the middle. Thus, P and R represent the sequences to be read at the ends and the fragment size is 2*k* + *s*. After adding adaptors and cDNA synthesis/PCR, we will have double-stranded products of the form [5′-PQR-3′/3′-pqr-5′] where p, q, r represent the reverse complement sequences of P, Q, R, respectively. Continued PCR amplification of these products will retain the same form because the copy of 5′-PQR-3′ is 3′-pqr-5′ and vice versa. These products are strand separated and captured in the flow cell and bridge amplified, recreating the [5′-PQR -3′/3′-pqr-5′] ensemble, but in single strand forms. After this step, each cluster on the flow cell contains a 5′-PQR-3′ string and 3′-pqr -5′ string, anchored at the 5′ end. Paired-end sequencing from this cluster extends from the 3′ ends of each string with the result that reads are obtained from the R template (as r) and the p template (as P). In sum, from the original fragment, 5′-PQR-3′, we obtain one 5′ end read of P in the sense direction and one 3′ end read of R in the antisense direction.

Suppose we have an mRNA and an internal fragmentation break point X. Around this breakpoint we have the left fragment, 5′-NNNX-3′ and the right fragment 5′-XNNN-3′, where X indicates the break position. Paired-end sequencing will create a sense read starting from X for the right fragment and an antisense read starting from X for the left fragment. Therefore, around X we will see both sense and antisense reads offset from each other by *k* bases. But random fragmentation means there will be other breakpoints, say at position X + 1, that will generate another pair of sense and antisense reads. The sum of such random fragmentation should be such that, on average, we will see equal numbers of sense reads and antisense reads around any internal position X. However, at the 5′ and 3′ end of the original mRNA, we can only have sense reads or antisense reads, respectively, because there will be no downstream or upstream fragment. Thus, an excess of sense reads or excess of antisense reads indicates ends of original mRNA.

Our aRNA amplification procedure preferentially produces template amplicons at the 3′ end of the original mRNA. Therefore, we concentrated on identifying 3′ UTR isoform variants using our Illumina RNA-seq data. For each gene, we computed the start position (defined as the left-most genomic coordinate of a read) of each sense and antisense read, assigning a directional read multiplicity value to each genomic position. To reduce random noise, we then computed a moving average of the read coverage for the sense and antisense direction, respectively, using a window size of 50 that is moved by 5 base pairs. The resulting moving window averages were used to compute a differential (Number of antisense reads − Number of sense reads) for each genomic position. The differential peaks generated in this manner were normalized by percentile height and the base positions under top 2 % peaks with a read depth of ≥10 were retained as candidate 3′ UTR end positions. For each gene, we computed the pairwise overlap in 3′ UTR ends of two samples as follows. Let A and B represent the samples. Then for each identified 3′ UTR end position in sample A, we scored a match if there was a 3′ UTR position in sample B within 200 base pairs of sample A’s position. Then pairwise overlap was defined as:

$$ d\;\left(A,B\right)=\left[\frac{\#\# Matches\; of\;A}{\# 3\mathit{\hbox{'}}UTR\; ends\; in\;A}+\frac{\# Matches\; of\;B}{\# 3\mathit{\hbox{'}}UTR\; ends\; in\;B}\right]\;/2 $$

Each identified 3′ UTR end position of every cell was compared with all of the other cells and a table was prepared that gives the fraction of cells of each cell type that has the peak. If in any cell type the gene was expressed in fewer than five cells, then that cell type was ignored for the gene. Then we identified three categories of genes using this table. First, genes that share all identified 3′ UTR positions were defined as those where, for all the expressed cell types, ≥80 % of the cells showed the 3′ UTR end and there were at least two different cell types involved. Second, genes for which no cells share the same set of 3′ UTR ends were defined as those for which the fraction was <0.6 for all putative 3′ UTR ends in all expressed cell types and there were at least two different cell types involved. Third, genes where there is evidence of cell-type-specific use of 3′ UTR ends were defined as those where there is a coherent 3′ UTR end (≥80 %) in at least one cell type which is absent or less coherent in other cell types.

### Data access

Raw RNA-sequencing data are archived in NCBI’s Gene Expression Omnibus [GEO: GSE56638].

## Additional files

Additional file 1:
**Supplemental tables. Table S1.** Dataset and RNA-sequencing statistics for each cell type. **Table S2.** RNA sequencing and quality control data for each single-cell sample. See "Materials and methods" for details about quality indicators used. **Table S3.** Summary of single-cell transcriptome characteristics. See "Results" for details on cell size adjustment. **Table S4.** Association between common expression and mutant phenotypes. Contingency tables show counts of genes with common expression and for remaining expressed genes (with at least one read in at least one sample for the indicated sample set), categorized as causing indicated mutant phenotype or not. Tables are shown for all significant pairwise associations (chi-square tests of independence with Bonferonni-corrected *p* values <0.05) and also for cardiomyocyte samples and mutant phenotypes. Phenotypic annotations were accessed from the Mammalian Phenotype Ontology on 23 June 2013 [47, 48]. Annotation IDs assigned to each mutant phenotypic category can be found in Table S7. **Table S5.** Within- and between-cell-type transcriptome variance computed on variance-stabilized frequencies. Only genes with an average relative frequency of greater than 6.3 × 10^−5^ across all cells are included. Variance stabilization: asin(2 × −1) − asin(−1). **Table S6.** Enrichment of Gene Ontology (GO) categories among variable genes identified by the F-statistic, variable genes identified by the outlier-sum statistic, and normally expressed genes by cell type. **Table S7.** Mammalian Phenotype Ontology annotations considered indicative of prenatal lethality or of tissue-specific mutant phenotypes [48]. Additional file 2: Figure S1. Dataset quality and transcriptome characteristics. **a, b** Single-cell samples were sequenced to a sufficient depth. **a** Pseudo-single-cell RNA-sequencing libraries with a range of sequencing depths, generated by randomly subsampling reads from single-cell RNA-sequencing libraries. We generated 100 pseudo-libraries for nine cells of each mouse cell type ranging from 0.5 million to 9.5 million uniquely aligning exonic reads. **b** Number of observed genes as a function of sequencing depth for single-cell dataset. **c** Complete-linkage hierarchical clustering of single-cell samples based on Euclidean distance between gene expression profiles using log_10_ zero-corrected normalized read counts for marker gene expression. **d, e** Pairwise correlations of zero-corrected normalized read counts for all detected genes (greater than zero reads in any sample) on a log_10_ scale. **d** Pairwise Pearson correlation coefficients for entire mouse dataset. Sample tissue or dilution input amount for a given row is indicated by the colored bar to the left of the heatmap and for a given column by the colored bar above the correlation heatmap. **e** Scatter plots of technical amplification replicates beginning with 10 picograms (pg) of total RNA. Upper triangle contains Pearson correlation coefficient. **f, g** Tissues differ in single-cell transcriptome characteristics. **f** The number of highly expressed genes comprising 50 % of reads by cell type. **g** The number of genes found only in a single cell by cell type. **h** The number of genes detected by cell type after correction for cell size. An adjusted detection threshold has been applied to each pyramidal neuron, removing all genes with expression below eight times the minimum observed relative frequency. The 100 picogram dilution replicates have been similarly corrected for tenfold and twofold differences in input RNA. Sample sizes, colors and abbreviations: brown adipocytes (*n* = 13, *brown*, *Adi*); cardiomyocytes (*n* = 19, *red*, *Car*); pyramidal neurons, cortex (*n* = 19, *dark blue*, *Pyc*); pyramidal neurons, hippocampus (*n* = 18, *light blue*, *Pyh*); serotonergic neurons, dorsal raphe (*n* = 22, *purple*, *Ser*); dilution controls starting with 100 pg (*n* = 9, *dark gray*, *100p*), 50 pg (*n* = 9, *gray*, *50p*), or 10 pg total RNA (*n* = 12, *light gray*, *10p*). Additional file 3: Dataset S1. Identified gene sets. Includes lists of private genes (genes observed in only one single-cell sample); universally common genes (genes observed in every single-cell sample); cell-type-specific common (genes abundantly expressed in every single-cell sample of a specific cell type, excluding universally common genes); 5 % most variable genes as identified by F-statistic; most variable genes by outlier-sum statistic; most normally expressed genes by Shapiro-Wilk statistic; and differentially variable genes across cell types. Lists are based on mouse data. Additional file 4: Figure S2. Accounting for technical characteristics of aRNA sequencing. **a–d** Selection of an expression level threshold for reliable quantification. Reported threshold read depths are average values across all biological samples. *% transcriptome* indicates the percent of the expressed transcriptome with expression levels greater than threshold, averaged across biological samples. In panels **a–c**, solid lines indicate median values across 500 neighbors by expression level for each group. **a** Fraction of replicates without detection of gene expression as a function of expression level. Vertical dotted line indicates threshold beyond which genes are reliably detected across replicates. **b** Variance across biological and dilution replicates as a function of expression level. Vertical dotted line indicates threshold beyond which genes’ median variation across biological replicates is at least twice the median variation observed across 10 picogram (pg) dilution replicates. **c** Normality of experimental variation as a function of expression level and amount of input RNA. Vertical dotted line indicates threshold beyond which Shapiro-Wilk *p* value >0.01 consistently across 10 pg dilution replicates. **d** Expression level (relative frequency) versus gene expression rank. Horizontal dotted line indicates estimated threshold beyond which frequency decreases more rapidly with gene rank in the 10 pg dilution controls. Solid lines represent individual samples. **e** Variation as a function of expression level across 10 pg dilution controls. Yellow line indicates median variation of 500 neighboring genes by expression level, which is used as an estimate of experimental variation as a function of expression level. **f** Scatter plots of gene traits and gene expression measurements. Mean read counts were calculated separately for each cell type and for dilution controls. **g** Experimental variation normality as a function of expression level for 10 pg dilution controls. Vertical dotted line indicates threshold beyond which Shapiro-Wilk *p* value >0.01 consistently across 10 pg dilution replicates. **h** F-statistic (ratio of single-cell variation to experimental variation) does not depend on gene GC content or gene length. Scatter plots of gene traits and F-statistic values. Mean F-statistic values were calculated separately for each cell type. Sample sizes, colors and abbreviations: 10 pg dilution replicates (*n* = 12, *light gray*, *10p*); 50 pg replicates (*n* = 9, *gray*, *50p*); 100 pg replicates (*n* = 9, *dark gray*, *100 p*); all single-cell samples (*n* = 91, *orange*, *All cells*). Additional file 5: Figure S3. A subset of genes demonstrates variable expression in each examined tissue type. **a–i** Scatter plots of F-statistic as a function of gene expression level for each experimental group. Vertical dashed line indicates quality control threshold used for variation analysis. Genes with relative frequencies below this threshold were not included in variation analysis. Horizontal dashed line indicates the top 5 % of included genes by F-statistic value for each cell type or dilution control. The F-statistic across 10 pg dilution controls is shown in black in panels **a**–**f. j** Scatter plot of average mRNA half-life in hours and outlier-sum statistic values across all single-cell samples. Sample sizes, colors and abbreviations: brown adipocytes (*n* = 13, *brown*, *Adi*); cardiomyocytes (*n* = 19, *red*, *Car*); pyramidal neurons, cortex (*n* = 19, *dark blue*, *Pyc*); pyramidal neurons, hippocampus (*n* = 18, *light blue*, *Pyh*); serotonergic neurons, dorsal raphe (*n* = 22, *purple*, *Ser*); all single-cell samples (*n* = 91, *orange*, *All cells*); 10 pg dilution replicates (*n* = 12, *black*, *10p*); 50 pg. dilution replicates (*n* = 9, *black*, *50p*); 100 pg dilution replicates (*n* = 9, *black*, *100p*).
