# Supplementary figures and images for: Deep sequencing reveals cell-type-specific patterns of single-cell transcriptome variation
Source: Genome Biol. 2015 Jun 9;16(1):122. doi: 10.1186/s13059-015-0683-4 (PMC4480509; doi:10.1186/s13059-015-0683-4)

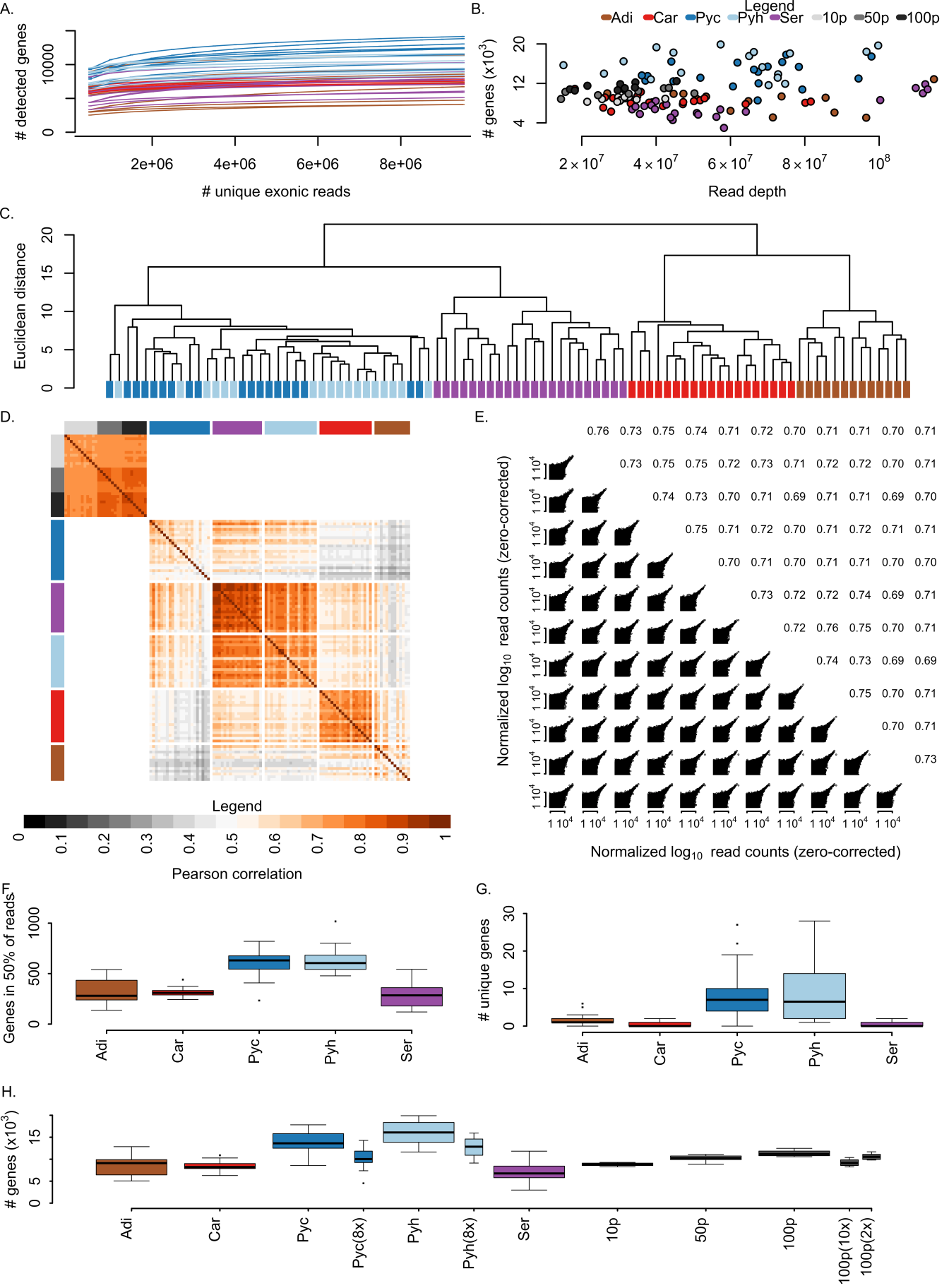

Supplement: Additional file 2: Figure S1. — Dataset quality and transcriptome characteristics. a, b Single-cell samples were sequenced to a sufficient depth. a Pseudo-single-cell RNA-sequencing libraries with a range of sequencing depths, generated by randomly subsampling reads from single-cell RNA-sequencing libraries. We generated 100 pseudo-libraries for nine cells of each mouse cell type ranging from 0.5 million to 9.5 million uniquely aligning exonic reads. b Number of observed genes as a function of sequencing depth for single-cell dataset. c Complete-linkage hierarchical clustering of single-cell samples based on Euclidean distance between gene expression profiles using log10 zero-corrected normalized read counts for marker gene expression. d, e Pairwise correlations of zero-corrected normalized read counts for all detected genes (greater than zero reads in any sample) on a log10 scale. d Pairwise Pearson correlation coefficients for entire mouse dataset. Sample tissue or dilution input amount for a given row is indicated by the colored bar to the left of the heatmap and for a given column by the colored bar above the correlation heatmap. e Scatter plots of technical amplification replicates beginning with 10 picograms (pg) of total RNA. Upper triangle contains Pearson correlation coefficient. f, g Tissues differ in single-cell transcriptome characteristics. f The number of highly expressed genes comprising 50 % of reads by cell type. g The number of genes found only in a single cell by cell type. h The number of genes detected by cell type after correction for cell size. An adjusted detection threshold has been applied to each pyramidal neuron, removing all genes with expression below eight times the minimum observed relative frequency. The 100 picogram dilution replicates have been similarly corrected for tenfold and twofold differences in input RNA. Sample sizes, colors and abbreviations: brown adipocytes (n = 13, brown, Adi); cardiomyocytes (n = 19, red, Car); pyramidal neurons, cortex (n = 19, dar [file 13059_2015_683_MOESM2_ESM.pdf]

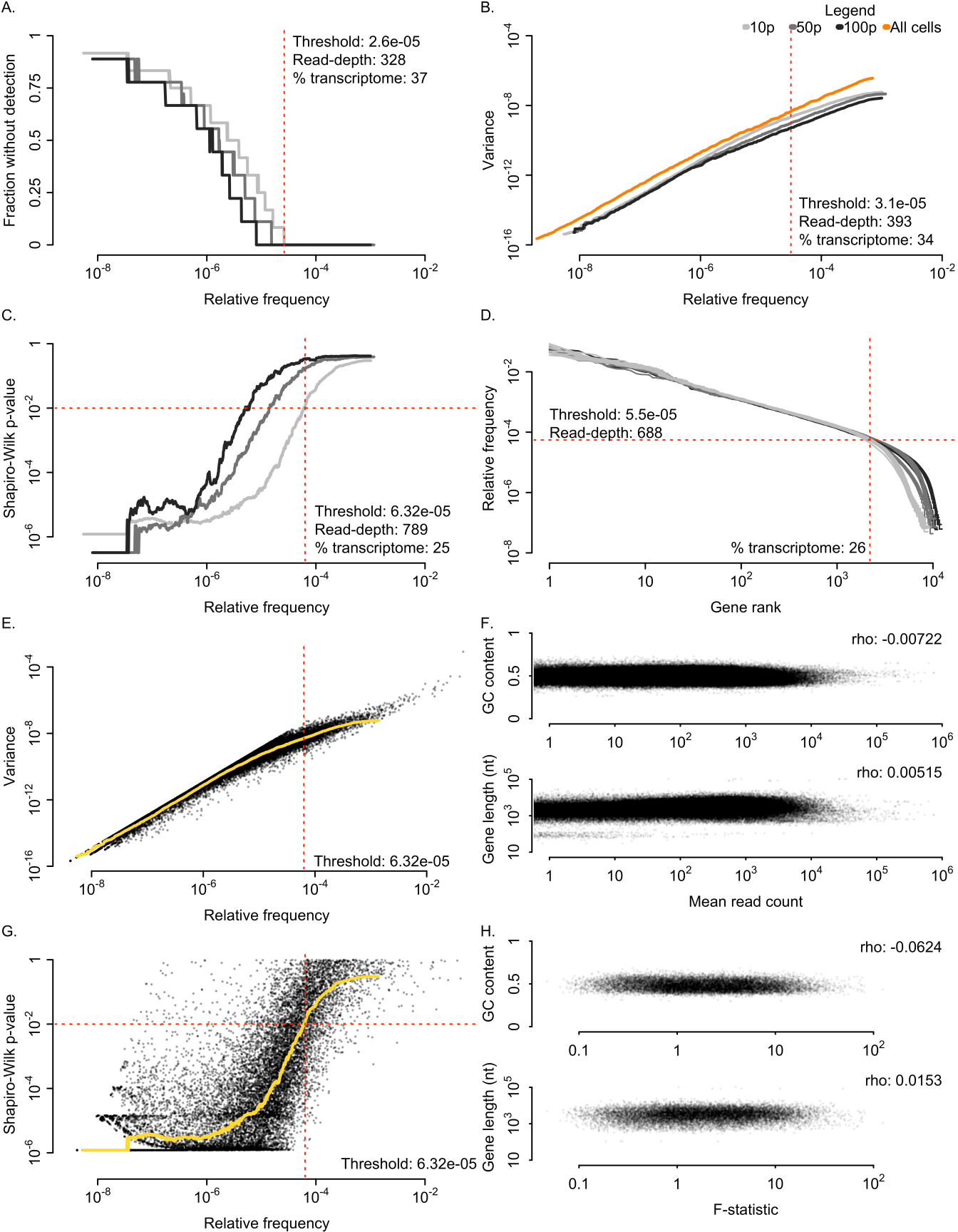

Supplement: Additional file 4: Figure S2. — Accounting for technical characteristics of aRNA sequencing. a–d Selection of an expression level threshold for reliable quantification. Reported threshold read depths are average values across all biological samples. % transcriptome indicates the percent of the expressed transcriptome with expression levels greater than threshold, averaged across biological samples. In panels a–c, solid lines indicate median values across 500 neighbors by expression level for each group. a Fraction of replicates without detection of gene expression as a function of expression level. Vertical dotted line indicates threshold beyond which genes are reliably detected across replicates. b Variance across biological and dilution replicates as a function of expression level. Vertical dotted line indicates threshold beyond which genes’ median variation across biological replicates is at least twice the median variation observed across 10 picogram (pg) dilution replicates. c Normality of experimental variation as a function of expression level and amount of input RNA. Vertical dotted line indicates threshold beyond which Shapiro-Wilk p value >0.01 consistently across 10 pg dilution replicates. d Expression level (relative frequency) versus gene expression rank. Horizontal dotted line indicates estimated threshold beyond which frequency decreases more rapidly with gene rank in the 10 pg dilution controls. Solid lines represent individual samples. e Variation as a function of expression level across 10 pg dilution controls. Yellow line indicates median variation of 500 neighboring genes by expression level, which is used as an estimate of experimental variation as a function of expression level. f Scatter plots of gene traits and gene expression measurements. Mean read counts were calculated separately for each cell type and for dilution controls. g Experimental variation normality as a function of expression level for 10 pg dilution controls. Vertical dotted line indicates threshold beyond w [file 13059_2015_683_MOESM4_ESM.pdf]

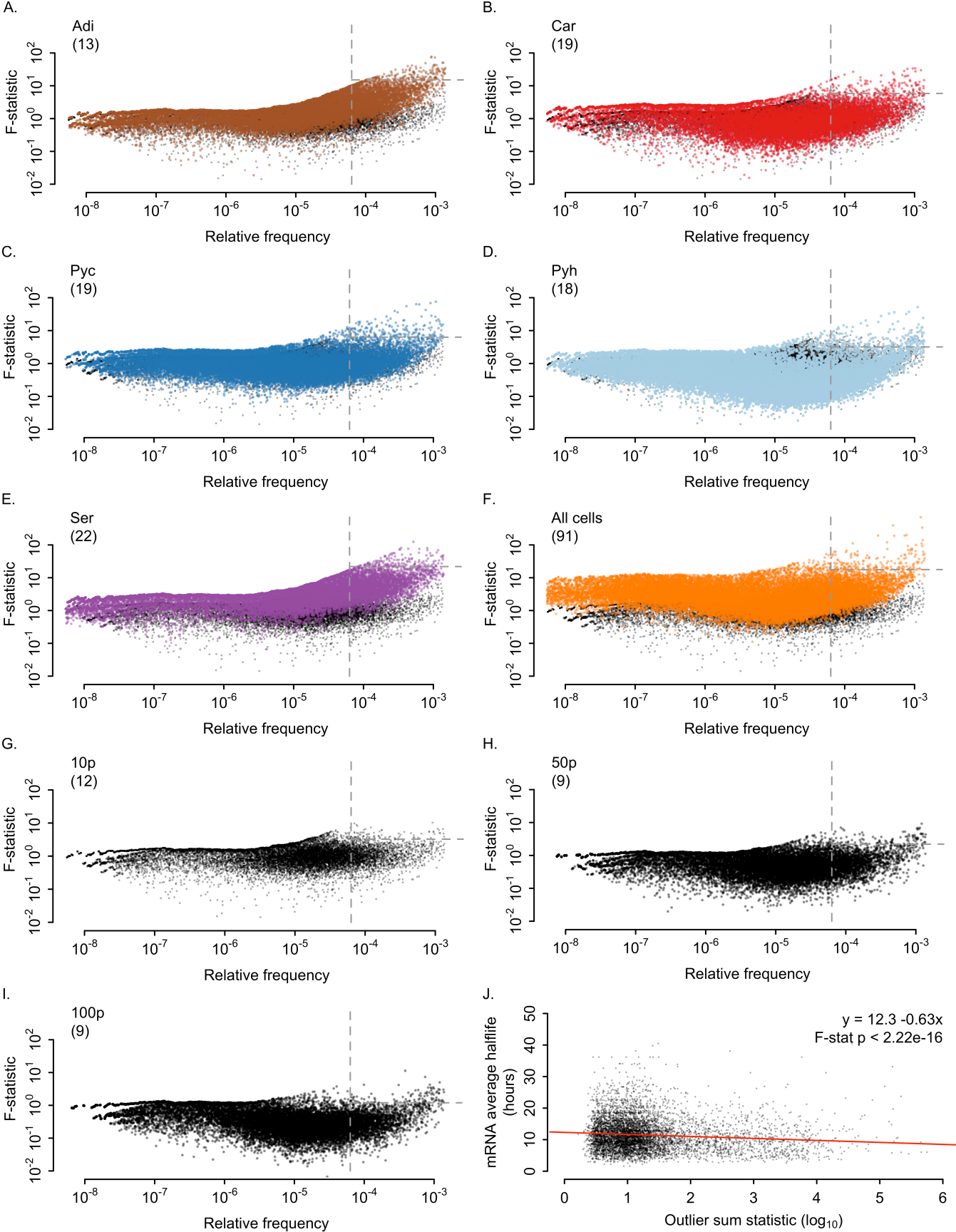

Supplement: Additional file 5: Figure S3. — A subset of genes demonstrates variable expression in each examined tissue type. a–i Scatter plots of F-statistic as a function of gene expression level for each experimental group. Vertical dashed line indicates quality control threshold used for variation analysis. Genes with relative frequencies below this threshold were not included in variation analysis. Horizontal dashed line indicates the top 5 % of included genes by F-statistic value for each cell type or dilution control. The F-statistic across 10 pg dilution controls is shown in black in panels a–f. j Scatter plot of average mRNA half-life in hours and outlier-sum statistic values across all single-cell samples. Sample sizes, colors and abbreviations: brown adipocytes (n = 13, brown, Adi); cardiomyocytes (n = 19, red, Car); pyramidal neurons, cortex (n = 19, dark blue, Pyc); pyramidal neurons, hippocampus (n = 18, light blue, Pyh); serotonergic neurons, dorsal raphe (n = 22, purple, Ser); all single-cell samples (n = 91, orange, All cells); 10 pg dilution replicates (n = 12, black, 10p); 50 pg. dilution replicates (n = 9, black, 50p); 100 pg dilution replicates (n = 9, black, 100p). [file 13059_2015_683_MOESM5_ESM.pdf]
